# Systematic Literature Review of Hearing Preservation Rates in Cochlear Implantation Associated With Medium- and Longer-Length Flexible Lateral Wall Electrode Arrays

**DOI:** 10.3389/fsurg.2022.893839

**Published:** 2022-07-01

**Authors:** Paul H Van de Heyning, Stefan Dazert, Javier Gavilan, Luis Lassaletta, Artur Lorens, Gunesh P Rajan, Henryk Skarzynski, Piotr H Skarzynski, Dayse Tavora-Vieira, Vedat Topsakal, Shin-ichi Usami, Vincent Van Rompaey, Nora M Weiss, Marek Polak

**Affiliations:** ^1^ Department of Otorhinolaryngology and Head and Neck Surgery, Antwerp University Hospital, Department of Translational Neurosciences, University of Antwerp, Antwerp, Belgium; ^2^ Department of Otorhinolaryngology-Head and Neck Surgery, Ruhr-University Bochum, St. Elisabeth University Hospital Bochum, Bochum, Germany; ^3^ Hospital Universitario La Paz, Institute for Health Research (IdiPAZ), Madrid, Spain; ^4^ Biomedical Research Networking Centre on Rare Diseases (CIBERER), Institute of Health Carlos, III, (CIBERER-U761), Madrid, Spain; ^5^ World Hearing Center, Institute of Physiology and Pathology of Hearing, Kajetany, Poland; ^6^ Department of Otolaryngology, Head and Neck Surgery, Luzerner Kantonsspital, Luzern; ^7^ Department of Health Sciences and Medicine, University of Lucerne, Luzern, Switzerland; ^8^ Otolaryngology, Head & Neck Surgery, Division of Surgery, Medical School University of Western Australia, Perth, Australia; ^9^ Heart Failure and Cardiac Rehabilitation Department, Medical University of Warsaw, Warsaw, Poland; ^10^ Institute of Sensory Organs, Kajetany, Poland; ^11^ Audiology Department, Fiona Stanley Fremantle Hospitals Group, Perth, WA, Australia; ^12^ Department of Otorhinolaryngology and Head and Neck Surgery, University Hospital Brussels, Vrije Universiteit Brussel, Brussels Health Campus, Belgium; ^13^ Department of Hearing Implant Sciences, Shinshu University School of Medicine, Matsumoto, Nagano, Japan; ^14^ Department of Electrophysiology, R&D, MED-EL, Innsbruck, Austria

**Keywords:** hearing preservation cochlear implantation, electrode length, electric-acoustic stimulation, flex 24, flex 28, flexSoft

## Abstract

**Background:**

The last two decades have demonstrated that preoperative functional acoustic hearing (residual hearing) can be preserved during cochlear implant (CI) surgery. However, the relationship between the electrode array length and postoperative hearing preservation (HP) with lateral wall flexible electrode variants is still under debate.

**Aims/Objectives:**

This is a systematic literature review that aims to analyze the HP rates of patients with residual hearing for medium-length and longer-length lateral wall electrodes.

**Method:**

A systematic literature review methodology was applied following the Preferred Reporting Items for Systematic Reviews and Meta-Analysis (PRISMA) recommendations to evaluate the HP rates of medium-length and longer-length lateral wall electrodes from one CI manufacturer (medium length FLEX 24, longer length FLEX 28 and FLEX SOFT, MED-EL, Innsbruck, Austria). A search using search engine PubMed (https://www.ncbi.nlm.nih.gov/pubmed/) was performed using the search terms “hearing preservation” or “residual hearing” and “cochlear implant” in “All fields.” Articles published only in English between January 01, 2009 and December 31, 2020 were included in the search.

**Results:**

The HP rate was similar between medium-length (93.4%–93.5%) and longer (92.1%–86.8%) electrodes at 4 months (*p* = 0.689) and 12 months (*p* = 0.219). In the medium-length electrode group, patients under the age of 45 years had better HP than patients above the age of 45 years.

**Conclusions:**

Both medium-length and longer electrode arrays showed high hearing preservation rates. Considering the hearing deterioration over time, implanting a longer electrode at primary surgery should be considered, thus preventing the need for future reimplantation.

## Introduction

The current technological and clinical state-of-the-art cochlear implant (CI) surgery is the result of the last 30 years of translational research between CI manufacturers and clinicians (1). Today, the indication for CI has been expanded to patients who have residual to normal hearing in the low-frequency (LF) region and ski-sloped mid- and high-frequency hearing loss but cannot benefit from a conventional hearing aid. This audiological indication is also called partial deafness (2). The region of functional LF acoustic hearing expanded from 500 to 1,500 Hz (3).

In 1997, Von Illberg et al. introduced the concept of combining electric and acoustic stimulation (EAS) as a mode of treating patients with nonprogressive partial deafness, demonstrating the feasibility of performing CI surgery without totally losing the residual hearing (4). The initial EAS study used insertion of a short electrode of <20 mm and postoperative acoustic amplifying of LF hearing with conventional hearing aids (HA) in combination with mid- and high-frequency electrical stimulation. The first multicenter EAS studies applying these short insertion depths of 19 mm demonstrated that the preservation, even partially, of residual hearing improved speech understanding in comparison to electrical stimulation only (5). This application was also successfully implemented by Gantz et al., confirming the feasibility of HP surgery and the application of EAS (6). In 2003, Skarzynski et al. demonstrated the combination of nonamplified LF hearing with electric stimulation for the subgroup of patients with normal or close-to-normal LF hearing in the implanted ear (2, 3).

These studies encouraged the development of hearing preservation (HP) surgical techniques, such as the application of corticosteroids, slow electrode insertion, avoidance of blood and bone dust entering the scala tympani, and avoidance of perilymph aspiration (7).

From then on, specific electrode arrays dedicated for EAS were developed with medium-length electrodes of 24 mm, reporting encouraging HP rates (8–10). To assess the effect of this CI surgery on HP, the Hearring group developed a calculation method of residual hearing and classification of HP (11).

Later on, hearing preservation with long 28 mm and 31 mm electrodes was reported (12–17).

The rationale for these long electrodes was to provide electrical coverage to the entire spiral ganglion in case residual hearing was lost over time (12, 17), as deeper insertion leads to higher speech recognition outcomes (18, 19).

Moreover, placing the electrode in the LF residual hearing region overlapping electric and acoustic stimulation has been shown to achieve the most benefit with EAS (20). With preserved residual hearing, the estimating electrode channels placed in the acoustic region could be switched off and progressively reactivated without the need for reimplantation surgery if the hearing declines (21).

The rationale for implanting longer electrodes in patients with partial deafness to achieve EAS is controversial; hence, this systematic literature review compares medium-length electrodes with longer electrodes with regard to their respective hearing preservation outcomes.

## Methods

### Search Strategy

A systematic literature review methodology was applied following the Preferred Reporting Items for Systematic Reviews and Meta-Analysis (PRISMA) (22) recommendations to evaluate the HP rates of the following flexible lateral wall electrodes from one particular CI. A search using the search engine PubMed (https://www.ncbi.nlm.nih.gov/pubmed/) was performed using the search terms “hearing preservation” or “residual hearing” and “cochlear implant” in “All fields.” Articles published only in the English language between January 01, 2009 and December 31, 2020 were included in the review.

### Inclusion Criteria

Studies with pre- and postop audiograms were included in the study. Studies must report on the HP rates by applying the HEARRING group’s HP classification (11) with pre- and postoperative audiograms for calculating the HP rates, or in the case the studies did not report the HEARRING group’s HP classification, individual pre-op and post-op audiograms were necessary to calculate the HP.

Only studies reporting on the FLEX24 (flexible 24 mm electrode) or FLEX28/FLEXSOFT electrodes (flexible 28 and 31 mm electrode) (MED-EL, Medical Electronics, Innsbruck, Austria) were included in this review. For the FLEX24 studies, a minimum electrode insertion depth of 20 mm was required (to reach 360° of electrode insertion), and for the FLEX28/FLEXSOFT studies, a postoperative CT evaluation or operative report confirming full insertion of FLEX28 (all 12 contacts inserted) or a minimum of 28 mm insertion depth was required.

Only MED-EL FLEX electrodes were chosen due to their design similarities and to reduce the effect of electrode design differences on the HP result’s comparison.

Further on, the term medium-length electrode will be used for the 24-mm FLEX24 electrode, being designed to reach the first turn of the cochlea. Longer electrodes are those that always reach the second turn, irrespective of the cochlear size, and comprises the 28-mm FLEX28 and the 31-mm FLEXSOFT electrodes.

**Figure 1:**
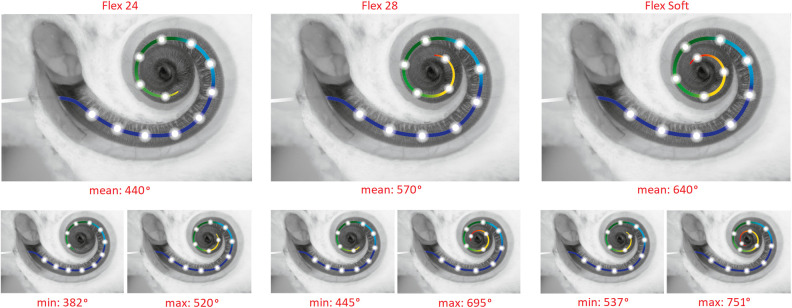
Electrode insertion ranges for Flex 24, Flex 28, and Flex Soft electrodes. To show the true data, only patients with hearing preservation and full insertion were included. The data are from the papers (22–27) (*n* = 163).

Mean electrode insertion angular depths by the three different electrode lengths are depicted in Figure 1 based on six studies (23–28).

The review included only patients with LF preoperative pure tone average (PTA) measured ≤65 dBHL across frequencies from 125 to 750 Hz with a minimum follow-up of 4 months and 12 months after cochlear implantation. The motivation for these audiological criteria was that such patients are considered EAS candidates.

### Hearing Preservation (HP) Rate Calculation

The HEARRING group (11, 29) proposed the following formula for qualitative HP classification: Relative change = ((PTA_post_-PTA_pre_) / (PTA_max_-PTA_pre_))

where PTA_post_ is the pure tone average measured postoperatively, PTA_pre_ is the pure tone average measured pre-operatively, and PTA_max_ is the maximal output limits of the audiometer. PTA is calculated for all characteristic frequencies attempted to measure the audiogram (125–8,000 Hz).

The classification based on this equation is independent of the initial hearing level and can be used for all CI users with measurable preoperative residual hearing (PTA: 0–120 dB). The misleading tendency of poorer preoperative hearing showing better postoperative HP results is eliminated because the classification is scaled to the preoperative audiogram. The equation also presents the relative change as a percentage of hearing loss, which is a user-friendly concept. The intervention-induced hearing loss is converted to hearing preservation (HP) by calculating 100%−relative change in %:

$$S=(1-\left(\left(\mathrm{PT}A_{\;\mathrm{post}}-\mathrm{PT}A_{\;\mathrm{pre}}\right)/\left(\mathrm{PT}A_{\max}-\mathrm{PT}A_{\;\mathrm{pre}}\right)\right)*100\%$$

where S is the preservation numerical scale.

In our case, if HP classification was not calculated, PTA_max_ was substituted with 110 dB HL. Finally, the numerical scale is converted to a categorical scale for easy reporting. The categorization is defined in Table 1.

**Table 1:** Scale of HP classification.

Percent of residual hearing preserved	Classification
>75%	Complete HP
>25%–75%	Partial HP
0%–25%	Minimal HP
No measurable hearing	No hearing

According to this classification, we defined HP if the patient reached complete or partial hearing preservation.

### Data Extraction/Retrieval

HP is evaluated in those subjects when the postoperative (audiogram) PTA measured is ≤65 dBHL across all frequencies from 125 Hz to 750 Hz. If no measurements at 125 Hz were provided, we assume that the 125 Hz threshold is the same as for 250 Hz. If 750 Hz was not measured and subjects were measured at 500 Hz and 1,000 Hz, linear approximation was performed to obtain the 750 Hz threshold. We extracted the number of patients reported with HP when they fell under the defined HP to calculate the HP rates with each electrode length taken for analysis (i.e., number of patients with HP/total number of patients implanted).

The HP rates at a minimum of 4-month and 12-month follow-up were obtained in all patients fulfilling the minimum preoperative LF PTA of 65 dBHL. The overall HP rate was the HP rate with all subjects included. In all evaluations, it was at a minimum 4-month follow-up.

Data were collected on the surgical approach, specifically round window (RW) or cochleostomy. Some clinicians use the term “extended round window” approach; however, as this information is not available in all studies, this surgical approach was included in the RW approach. In general, an RW approach was defined as the approach used when the electrode was inserted via the round window membrane and in cases when the RW approach required an anterior-inferior extension to insert the electrode due to either the size of the round window, the insertion angle, or the electrode diameter.

Data from studies that were from clinical trials were reported separately. Prospective studies have more control over the subjects and data generation as compared to retrospective studies.

Additionally, we calculated and reported the complete HP rates. Complete HP rates were calculated by selecting the number of patients with complete HP divided by the total number of patients implanted with CI.

In addition, the age at implantation was assessed as to whether it plays an important role in HP. For this reason, subjects were split into two groups: one group up to and including 45 years of age and another group 45 years of age and above at the time of surgery. The age of 45 years was selected as it was an approximate mean in both groups, the group of patients implanted with a medium-length electrode and the group of patients implanted with a longer electrode.

### Statistical Analysis

For the given pairwise comparisons, common statistical tests for significant differences were performed as implemented in STATISTICA 14.0 (TIBCO Software Inc. (2020) Data Science Workbench, version 14. http://tibco.com). A criterion of *α* = 0.05 for the comparisons was set. The criterion for statistical power was >0.9. Power calculations for two proportions, Z-test have been done in STATISTICA 14.0 to estimate, which min. difference would have been detected as significant with 90% probability (Power).

### Risk of Bias Assessment

**Figure 2:**
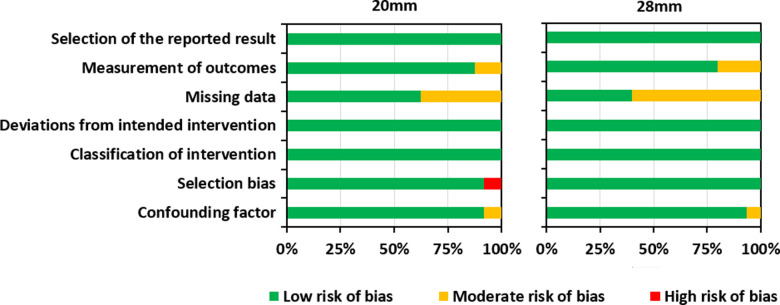
Results of the risk of bias assessment for both groups.

Risk of bias assessment helps to establish transparency of evidence synthesis results and findings. Evidence syntheses strive to eliminate bias in the findings. Therefore, the risk of bias was independently assessed by the fourth and the last authors (LL and MP). Included studies were assessed using the Risk of Bias in Non-randomized Studies of Interventions (ROBINS-I) Tool. (30). This tool contains seven items judging the risk of bias due to confounding, study participant selection, classification of interventions, deviations from intended intervention, missing data, measurement of outcomes, and selection of reported results. Each of the seven items in included studies was judged low, moderate, or high risk. Results of the risk of bias assessment were graphically summarized using Microsoft Excel (Figure 2) (https://www.microsoft.com/en-us/microsoft-365/excel). Confounding factors were the electrode type/insertion depth, surgical approach, and age at implantation.

## Results

### Search Results

**Figure 3:**
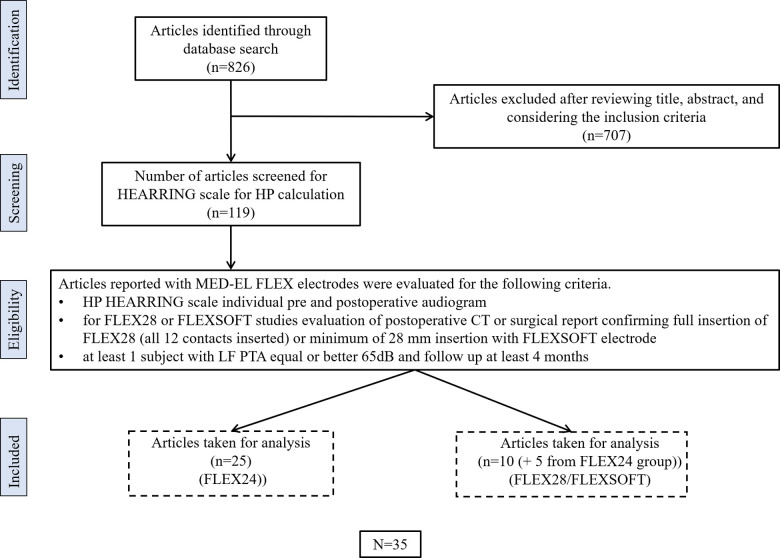
PRISMA flow chart detailing the selection of literature for inclusion in the study.

Thirty-three peer-reviewed publications reporting on HP with the FLEX24 or FLEX28/FLEX SOFT electrodes were identified using the search strategy. Figure 3 details the numbers of publications identified, screened, and eligible for inclusion in the analyses.

### Medium-Length Electrode FLEX24

A total of 25 peer-reviewed articles published between 2009 and 2020 reporting on HP were identified from the database and are listed in Table 2. A total of 304 cases were implanted with the FLEX24 electrode. All the articles confirmed that the insertion depth was at least 20 mm inside the cochlea.

**Table 2:** Studies that reported on the number of HP patients implanted with FLEX24 electrodes

No.	Study	Surgical approach	HP at 4-month follow-up	HP at 12 month follow-up
1	Gstöttner et al. (2009)	RW (*n* = 7) Cochl (*n* = 2)	9/9	9/9
2	Adunka et al. (2010)	Cochl (*n* = 10)	9/10	0/0
3	Arnoldner et al. (2011)	Cochl (*n* = 3) RW (*n* = 1)	3/4	3/3
4	Helbig et al. (2011)*	Cochl (*n* = 13)/RW (*n* = 4)	17/17	17/17
5	Erixon et al. (2012)	RW (*n* = 12)	12/12	8/8
6	Tamir et al. (2012)	RW (*n* = 4)	3/4	0/0
7	Rajan et al. (2012)	RW (*n* = 9)	9/9	9/9
8	de Carvalho et al. (2013)	RW (*n* = 3)/Cochl (*n* = 1)	3/4	3/4
9	Nordfalk et al. (2014)	RW (*n* = 5)	5/5	0/0
10	Santa Maria et al. (2013)	Cochl (*n* = 14)	12/14	12/14
11	Adunka et al. (2013)	RW (*n* = 10)/Cochl (*n* = 8)	16/18	16/18
12	Mertens et al. (2014)	Cochl (*n* = 4)	4/4	3/4
13	Usami et al. (2014)*	RW (*n* = 25)	25/25	25/25
14	Guimaraesa et al. (2014)	RW (*n* = 16)/Cochl (*n* = 3)	17/19	17/19
15	Bruce et al. (2014)	RW (*n* = 1)/ Cochl (*n* = 1)	2/2	1/1
16	Moteki et al. (2014)	RW (*n* = 4)	2/2	2/2
17	Mahmoud et al. (2014)	RW (*n* = 5)	5/5	5/5
18	Suhling et al. (2016)	RW (*n* = 10)	9/10	9/10
19	Pillsbury et al. (2018)*	RW (*n* = 51)/Cochl (*n* = 16)	61/67	61/67
20	Rader et al. (2018)	RW (*n* = 11)	11/11	5/5
21	Skarzynski et al. (2019)	RW (*n* = 8)	8/8	8/8
22	Thompson et al. (2019)	Cochl (*n* = 1)	1/1	1/1
23	Skarzynski et al. (2019)	RW (*n* = 11)	11/11	11/11
24	Schart-Moren et al. (2020)	RW (*n* = 15)	16/16	16/16
25	Sprinzl et al. (2020)	RW (*n* = 16)	13/16	9/10
			**284** **/** **304**	**245** **/** **262**

*Cochl: cochleostomy; RW: round window; 0 denotes no patients were available for the long-term follow-up. An asterisk indicates that the study was based on a clinical trial. The n indicates the total number of cases in the study*. *References in Appendix 1*.

**Figure 4:**
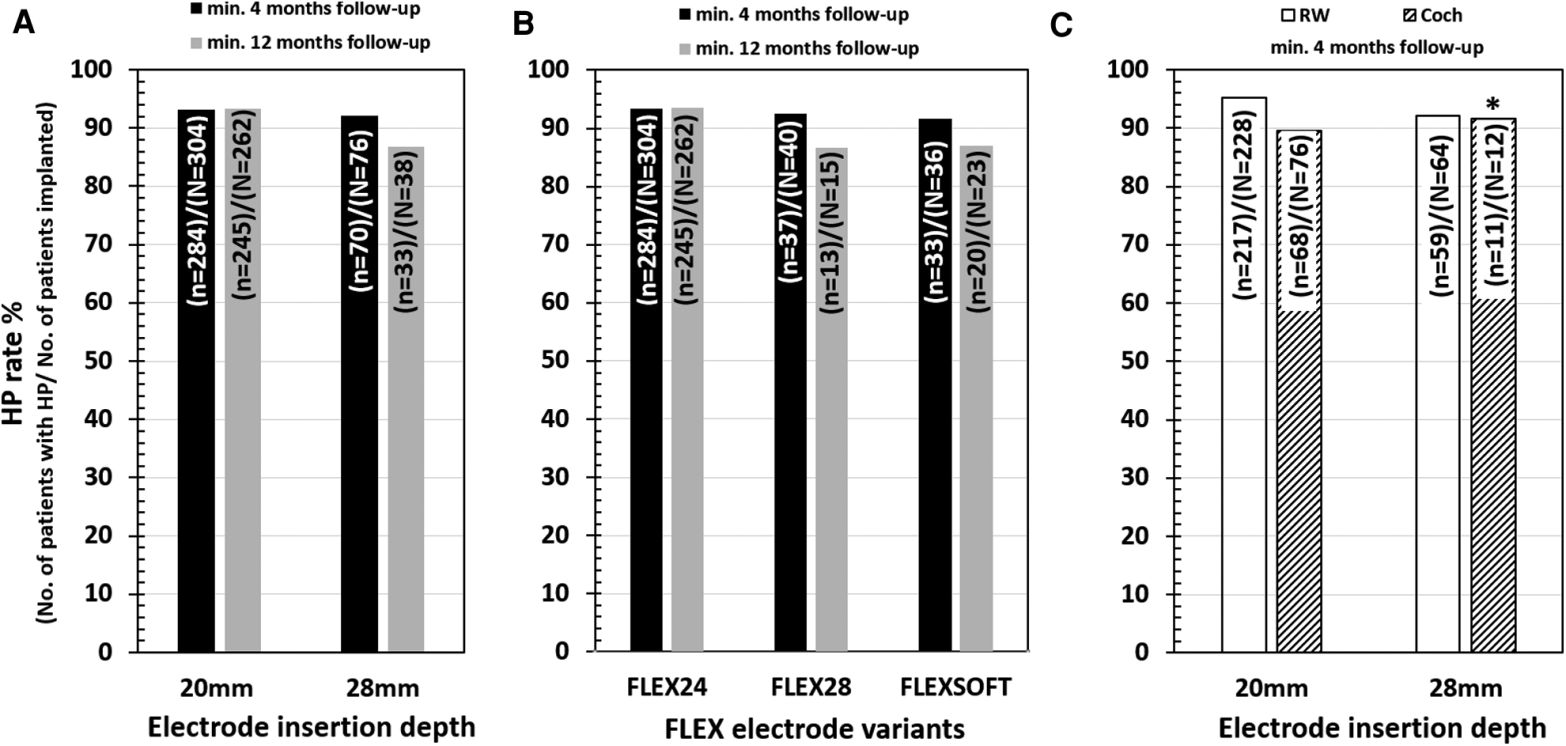
HP rates for two different electrode insertion depths (**A**), of all three flex electrode variants (**B**) and at a minimum of 4-month and 12-month postoperative follow-up. HP rates for two different electrode insertion approaches at a minimum of 4-month follow-up (**C**). The “*N*” refers to the total number of patients implanted, and the “*n*” refers to the number of patients with HP. The 20 mm insertion depth refers to the medium-length FLEX24 implanted group, and over 28 mm of insertion depth refers to the longer FLEX28 and FLEXSOFT implanted groups. * points to the small number of patients in the cochleostomy group implanted with longer electrodes.

Out of the 304 cases implanted with the FLEX24 electrode, 284 had HP, giving an overall HP rate of 93.4%. Within this group, 20 cases with minimum or no hearing were followed-up to 4 months and 12 months postoperatively. The HP rate with FLEX24 in the 4-month group was 93.4% and 93.5% in the 12-month group at follow-up postoperatively (Figures 4A, B).

Seventy-six cases were implanted using the cochleostomy approach, and the remaining 228 cases were implanted with the RW approach of electrode insertion. In the cochleostomy patients, the HP rate was 89.5% (68 of 76 patients) and 95.2% in the RW patients (217 of 228 patients), as shown in Figure 4C. The HP rate in patients implanted with RW surgical approach was 5.7% greater than that in cochleostomy patients (Figure 4C).

Among the 25 articles reviewed, 3 were prospective clinical trial studies. A total of 109 cases were implanted with the FLEX24 electrode, out of which 103 cases had HP, giving an overall HP rate of 94.5%. This gave an HP rate with FLEX24 in the 4-month group of 94.5% and 94.5% in the 12-month group at follow-up postoperatively.

### Longer-Length Electrodes FLEX28 and FLEXSOFT

A total of 15 peer-reviewed articles published between 2009 and 2020 reporting on HP were identified from the database and are listed in Table 3. Among these papers, five investigated FLEX24 subjects were already included in the FLEX24 studies. A total number of 76 cases were implanted with either the FLEX28 or the FLEXSOFT electrode.

**Table 3:** Studies that reported on the number of HP patients implanted with FLEX28 (F28) and FLEXSOFT (FS) electrodes

No.	Study	Approach	HP at 4-month follow-up	HP at 12-month follow-up
26	Helbig et al. (2011)	RW (*n* = 7) / Cochl (*n* = 1)	6/8 (FS)	6/8
27	Bruce et al. (2011)	Cochl (*n* = 5)	5/5 (FS)	0/0
28	Skarzynski et al. (2011)	RW (*n* = 9)	9/9 (FS)	9/9
3	Arnoldner et al. (2011)	Cochl (*n* = 1)	1 /1 (FS)	0/0
29	Rogers et al. (2012)	RW (*n* = 1)	1 /1 (FS)	0/0
30	Jayawardena et al. (2012)	RW (*n* = 1)	1 /1 (F28)	1/1
11	Mertens et al. (2014)	Cochl (*n* = 1)	1 /1 (FS)	1/1
12	Usami et al. (2014)	RW (*n* = 1)	1 /1 (FS)	1/1
14	Bruce et al. (2014)	Cochl (*n* = 4)	4 /4 (FS)	4/4
31	Nordfalk et al. (2016)	RW (*n* = 17)	11/12 (F28), 4/5 (FS)	0/0, 0/0
17	Suhling et al. (2016)	RW (*n* = 7)	5/7 (F28)	5/7
32	Jones et al. (2018)	RW (*n* = 4)	4 /4 (F28)	4/4
33	Moteki et al. (2019)	RW (*n* = 4)	4 /4 (F28)	0/0
			1 /1 (FS)	0/0
34	Sierra et al. (2019)	RW (*n* = 3)	3 /3 (F28)	3/3
35	Yoshimura et al. (2020)	RW (*n* = 9)	9 /9 (F28)	0/0
			**37/40 (F28)**	**13/15 (F28)**
			**33/36 (FS)**	**20/23 (FS)**

*Cochl: cochleostomy; RW: round window; 0 denotes no patients were available for the long-term follow-up. The n indicates the total number of cases in the study*. *References in Appendix 1*.

Of the 76 cases implanted with the FLEX28/FLEXSOFT electrode, 70 had HP as per the definition given in the Methods section, giving an overall HP rate of 92.1%. Within this group, 76 cases were followed-up for 4 months and 38 cases were followed-up for 12 months postoperatively. The HP rate with FLEX28/FLEXSOFT in the 4-month group was 90.8% and 86.8% in the 12-month group at follow-up post-operatively.

Of the 40 cases implanted with the FLEX28 electrode, 37 had HP, giving an overall HP rate of 92.5%. Within this group, 40 cases were followed-up for 4 months and 15 cases were followed-up for 12 months post-operatively. The HP rate with FLEX28 in the 4-month group was 92.5% and with FLEX28 in the 12-month group was 88.9% at follow-up postoperatively (Figure 4B).

Of the 36 cases implanted with the FLEXSOFT electrode, 33 had HP, giving an overall HP rate of 91.7%. Within this group, 36 cases were followed-up for 4 months and 23 cases were followed-up for 12 months postoperatively. The HP rate with FLEXSOFT in the 4-month group was 91.7% and with FLEXSOFT in the 12-month group was 86.9% at followup postoperatively (Figure 4B).

Twelve cases were implanted using the cochleostomy approach, and the remaining 64 cases were implanted with the RW approach of electrode insertion. In the cochleostomy patients, the HP rate was 91.7% (11 of 12 patients), and in the RW patients, the HP rate was 92.2% (59 of 64 patients), as shown in Figure 4C. Due to the small number of cochleostomy patients, a comparison of HP rates between cochleostomy and the RW approach could not be estimated in the FLEX28/FLEXSOFT cases.

None of the studies of the FLEX28/FLEXSOFT cases were based on prospective clinical trials.

**Figure 5:**
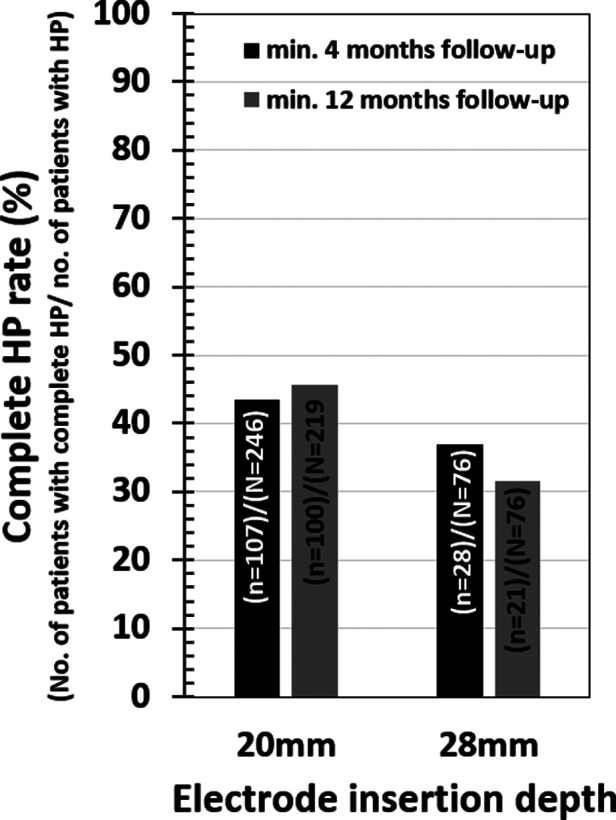
Complete HP rates for two different electrode insertion depths.

Among the selected papers, 19 studies related to the medium-length electrode and 15 studies related to the longer electrodes could identify information on the age of the patient at CI surgery and also complete HP. Table 4 Details the pairwise HP comparison statistical tests outcomes. None of the comparisons showed significant differences (all *p*-values > 0.05). At 4 month follow-up, 43.5% (107 from 246) of patients attained complete HP in the medium-length electrode group, while 36.8% (28 from 76) of patients attained complete HP in the longer electrode group (Figure 5). At 12-month follow-up, 45.7% (100 from 219) of patients attained complete HP in the medium-length electrode group, while 31.6% (12 from 38) of patients attained complete HP in the longer electrode group (Figure 5).

**Table 4:** The pairwise HP comparison statistical tests outcomes. * Comparisons are insufficient due to small sample size.

	28 vs. 20 mm; 4 months	28 vs. 20 mm; 12 months	28 vs. 20 mm for trials; 4 months	28 vs. 20 mm for trials; 12 months	Flex28 vs. Flex Soft; 4 months	Flex 28 vs. Flex Soft; 12 months	28 mm. cochl. Vs. RW	20 mm cochl. Vs. RW	28 vs. 20 mm, 4 months; complete HP	28 vs. 20 mm, 12 months; complete HP
P1 [%]	92.1	86.8	92.1	86.8	92.5	86.7	91.7	89.5	36.8	31.6
P2 [%]	93.4	93.5	94.5	94.5	91.7	87.0	92.1	95.2	43.5	45.7
N1	76	38	76	38	40	15*	12*	76	76	38
N2	304	262	109	109	36	23*	64	228	246	219
*p*-value	0.689	0.219	0.528	0.121	0.893	0.979	0.904	0.075	0.30	0.11

*None of the comparisons showed significant differences (all p-values > 0.05)*.

**Table 5:** The pairwise HP comparison statistical tests for complete HP and age comparison outcomes.

	±45yrs, 28 mm, 4 months, HP	±45 yrs, 28 mm, 12 months, HP	±45yrs, 28 mm, 4 months, complete HP	±45yrs, 28 mm, 12 months, complete HP	±45yrs, 20 mm, 4 months, HP	±45yrs, 20 mm, 12 months, HP	±45yrs, 20 mm, 4 months, complete HP	±45yrs, 20 mm, 12 months, complete HP	20 vs 28 mm, HP, 4 months, <45 yrs	20 vs 28 mm, HP, 12 months, <45 yrs	20 vs 28 mm, complete HP, 4 months, <45 yrs	20 vs 28 mm, complete HP, 12 months, <45 yrs	20 vs 28 mm, HP, 4 months, >45yrs	20 vs 28 mm, HP, 12 months, >45 yrs	20 vs 28 mm, complete HP, 4 months, >45 yrs	20 vs 28 mm, complete, 12 months, >45 yrs
P1 [%]	90.0	84.0	34.0	24.0	94.2	93.2	36.5	39.2	96,2	92.3	42.3	46.2	90.0	84.0	34.0	24.0
P2 [%]	96.2	92.3	42.1	46.2	96.3	95.2	51.2	54.8	96.3	95.2	51.2	54.8	94.2	93.2	36.5	39.2
N1	50	25	50	25	156	148	156	148	26	13	26	13	50	25	50	25
N2	26	13	26	13	82	62	82	62	82	62	82	62	156	148	156	25
p-value	0.278	0.425	0.481	0.173	0.449	0.575	**0**.**029**	**0**.**033**	0.965	0.717	0.424	0.568	0.361	0.225	0.743	0.108

None of the comparisons showed significant differences (all *p*-values > 0.05). For complete HP comparisons at 12-month follow-up, the medium length vs. longer electrode groups showed no statistical difference (*p* = 0.11).

**Figure 6:**
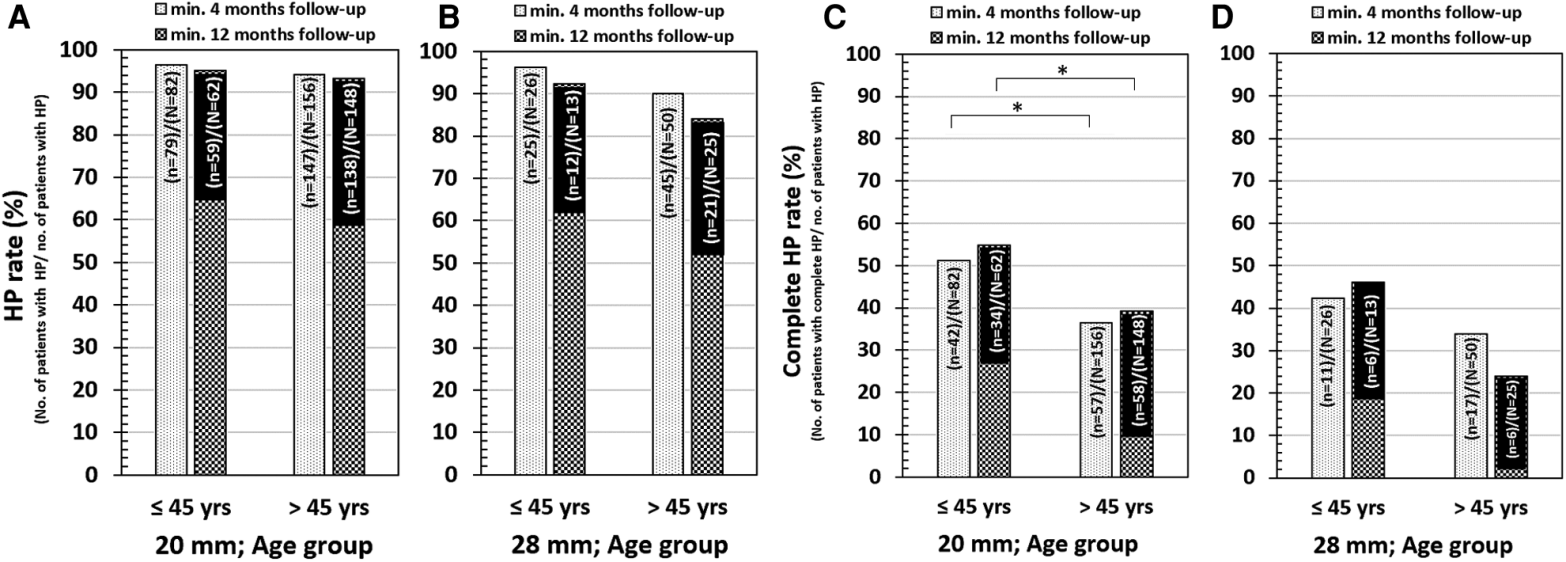
HP in 20 mm electrode insertion depth and for age below and above 45 years (**A**) and 28 mm electrode insertion depth for age below and above 45 years (**B**). Complete HP in 20 mm electrode insertion depth and for age below and above 45 years (**C**) and 28 mm electrode insertion depth for age below and above 45 years (**D**). The “*N*” refers to the total number of patients implanted, and the “*n*” refers to the number of patients with HP. The 20 mm insertion depth refers to the medium-length FLEX24 implanted group, and the 28 mm insertion depth refers to both longer FLEX28 and FLEXSOFT implanted groups.

For medium-length electrodes, HP in patients below 45 years at 4-month and 12-month follow-up was reached in 96.3% and 95.2% of cases, respectively (Figure 6A). HP in patients above 45 years of age at 4-month and 12-month follow-up was reached in 94.2% and 93.2% of cases, respectively (Figure 6A). Complete HP in patients below 45 years at 4-month and 12-month follow-up was reached in 51.2% and 54.8% of cases, respectively (Figure 6C). Complete HP in patients above 45 years of age at 4-month and 12-month follow-up was reached in 36.5% and 39.2%, respectively (Figure 6C). For longer electrodes, HP in patients below 45 years at 4-month and 12-month follow-up was reached in 96.2% and 92.3% of cases, respectively (Figure 6B). HP in patients above 45 years of age at 4-month and 12-month follow-up was reached in 90.0% and 84.0% of cases, respectively. Complete HP in patients below 45 years at 4-months and 12-month follow-up was reached in 42.3% and 46.2% of cases, respectively (Figure 6D). Complete HP in patients above 45 years of age at 4-month and 12-month follow-up was reached in 34.0% and 24.0% of cases, respectively (Figure 6D).

Table 5 details the pairwise complete HP and age comparison outcomes. For 20 mm insertion, patients below the age of 45 reached higher complete HP at the follow-up of 4 months and at the follow-up of 12 months (*p* < 0.05). The remaining comparisons were not significantly different (all *p*-values > 0.05). For age comparisons, complete HP at 12 months follow up for 28 mm insertion group, and the complete HP comparisons at 12 months follow up 20 mm vs 28 mm insertion we found quite remarkable difference in proportions, but still not significant (*p* = 0.173 and *p* = 0.108, respectively).

## Discussion

This systematic literature review (SR) shows high rates of stable HP for all flexible electrodes over time. The same applies to complete HP.

These results can be achieved using standardized surgical techniques such as the avoidance of perilymph aspiration, clean surgery avoiding blood and bone dust entering the scala tympani or sticking to the electrode, slow electrode insertion to avoid intracochlear pressure peaks, slow drilling with diamond burrs on the cochlea, and systemic and topical corticosteroids (7, 31).

Achieving HP in CI surgery seems to be closely associated with the development of dedicated atraumatic electrode arrays. Experimental data emphasizes that flexible electrodes are less traumatic than stiffer electrodes (32). The straight electrode array design is atraumatic due to the significant reduction of the risk of traumatic intracochlear electrode placement such as electrode tip fold-over and scalar deviation (33, 34). The data of this SR are standardized concerning a uniform slim flexible electrode design and show that functional HP can be achieved with longer electrodes. Consequently, this implies that the rate of HP might rather depend on the slim flexible design than on the length alone.

The first reports on EAS demonstrated lower HP rates because of the stiff electrodes used and the unrefined surgical techniques utilized at the time (4, 5). The potential perceived conflict of interest between optimization of cochlear coverage for electric stimulation vs. acoustic HP has led to ongoing research into preoperative hair cell and ganglion cell protection, spiral ganglion cochleometrics (1, 35), robotic drilling and insertion methods (36, 37), and perioperative electrophysiological monitoring (38) to further improve HP outcomes.

### Effects on HP

No significant differences in the HP rates between medium and long electrodes were demonstrated. This also applies to complete HP rates at the two time points investigated.

Although different consensus papers (7) advocate the RW approach for HP surgery, a distinct advantage over cochleostomy was not demonstrated in this SR. Besides the limited cases using cochleostomy, the fact that RW and extended RW were analyzed together is very likely to have influenced the outcome. Indeed, extending the RW niche by drilling anteroinferiorly into the RW rim causes additional intracochlear trauma. Extended RW drilling and promontory cochleostomy drilling cause a breach of the endoscalar epithelial lining, which is known to create new fibrous and bone tissue inside the cochlea, leading to hair cell and spiral ganglion loss and impairment of the cochlear micromechanics, such as the vibration of the basilar membrane (39). In addition, the trauma may also lead to hydrops (40).

Calculating the effect of age resulted in a significant difference in favor of patients under 45 years of age. This confirmed earlier reports showing better HP in children and younger adults (7, 27, 41–42).

### Progressive and Postoperative HL

Initially, EAS with medium electrodes was advocated for partial deafness, which was defined as a hearing deterioration of less than 15 dB in the LF in the last 2 years. Usami et al. (43, 44) identified several genetic hearing disorders with progressive LF residual hearing. Progressive hearing loss over time is a natural phenomenon in the majority of patients with residual hearing (45–47). Little is known about the individual evolution of the hearing decline in adults (48) and children with residual hearing (49).

Concerning long-term results for EAS patients using medium-length electrodes, postoperative residual hearing declines over time, and the deterioration of residual hearing of the implanted ear is related to the rate of progression in the contralateral ear (45, 46). A causative gene was detected that caused progressive hearing loss in half of the patients receiving EAS (42), suggesting that various genes are responsible in EAS patients.

As reported, patients with stable residual hearing can lose the residual hearing after CI surgery resulting in nonfunctional residual hearing levels.

Using a shorter electrode array can leave the patient with insufficient electric stimulation. This depends on the individual cochlear size and occurs when an electrode array that does not reach the second turn of the cochlea or the apical bulb of the spiral ganglion is situated at 650–690° (35, 50). Subsequently, in these situations, the electric activation over the full frequency range induces an important place–frequency mismatch (18, 27). Several publications provide evidence that deeper electrode insertion results in better speech understanding in cases where the residual hearing is lost or deteriorates significantly (19, 51).

### Solutions in the Case of Nonfunctional Hearing

Different strategies can be used in the case of nonfunctional HL after HP surgery with short or medium-length electrode arrays.

A possible approach is reimplantation. Fitzgerald et al. (52) reported that patients with partial deafness implanted with a hybrid cochlear electrode array of length 10 mm lost the residual hearing a few months after CI surgery. Reimplantation with a medium-length electrode in these patients improved the hearing performance from 59% to 86% and 7% to 36% at 3 months post-re-implantation (49). It is important to note that reimplantation with longer electrode arrays does not exclude the preservation of residual hearing. Jayawardene and Rajan demonstrated in a case series that HP could be achieved in reimplantation despite using longer electrodes (53).

A second solution was first presented by Lenarz et al. (54), partially implanting a longer electrode to a depth inside the cochlea where the residual hearing starts based on the preoperative calculation of the cochlear duct length and the patient-specific frequency map. This method was further developed in an experimental setting by Weiss et al. (55), who presented a technique that ensures a minimum of 12 electrode contacts within the cochlea. With this surgical technique, the partially implanted electrode is further advanced inside the cochlea through the external ear canal, requiring a minor second-stage surgery, thus providing electric coverage to the entire frequency range and without the need for a new implant (54). However, this procedure requires that the electrode is not fixed in the posterior tympanotomy at initial surgery with the potential risk of electrode slippage out of the cochlea (34).

A third way was published by Yoshimura et al., who reported implantation of a longer electrode in patients with LF hearing, with the concept of deactivating the apical channels placed in the LF hearing initially until the LF hearing deteriorated over time (28). Polak et al. demonstrated the possibility of estimating electrode channels placed in the acoustic region (21). This concept offers the possibility to reactivate those apical channels placed in the LF hearing region without any frequency shifting and change of the psychoacoustic thresholds of the basal electrode channels and thus minimizes the time adjustment, giving the possibility to provide electrical stimulation over the complete frequency range without the need for reimplantation surgery. It is worth mentioning that many CI patients are reluctant to change their electrical map once they are accustomed to a stable and satisfactory map; here, it is also important to note that it can take up to 1 year before the benefits of extended remapping translate into measurable improvements of speech recognition (56). This approach relies on an atraumatic insertion of a long flexible array that provides a larger cochlear coverage in the case of hearing deterioration or loss of residual hearing.

### Limitations of the SR

Although some trends were noticeable, the number of cases was too small to draw any final conclusion as to whether or not medium electrode insertions are different from longer electrode insertions regarding HP. Not all the studies reported their data in a similar way. Furthermore, many studies were retrospective, with low data quality. It is important to note that the results of the prospective studies did not differ from the retrospective studies, reporting comparable high HP rates. However, there were no prospective registered studies on 28-mm and 31.5-mm flexible electrodes

The lack of significant differences between the electrode lengths may be due to the following confounding aspects. First, the individual sizes of the cochlea were not taken into account. Due to the large cochlear size variation of up to 50%, the insertion angles will vary accordingly, despite using the same electrode length (Figure 1). Subsequently, medium-length electrodes may reach a similar insertion depth in a small cochlea as longer electrodes implanted in a longer cochlea. The intracochlear volume has a large influence on electrode insertion forces and intracochlear pressure changes (57), and these effects may mask the actual impact of the electrode lengths. This study emphasizes the importance of slim electrodes and slow insertion speeds. To eliminate this bias, standardized prospective studies with preoperative cochlear size assessment are required. Second, in this SR, cases were not stratified according to their hearing loss pattern, e.g., stable hearing vs. natural progressive sensorineural hearing loss. This might confound the effects of insertion length differences. A slower decline of hearing becoming significant over longer periods of time has been reported (46, 49). Other studies reported a yearly decline of 3% over a 10-year period using standard electrodes with 19 mm insertion (56). However, since this SR focused on the effect of HP during surgery and no statistical differences were found between 4-month and 12-month follow-up, this effect is considered minor.

In addition, the Covid pandemic, with its effects on restriction on clinical studies, resulted in limiting the SR till 2020 (58).

A differentiation between the RW approach and the extended RW approach could not be made and thus does not demonstrate the effect of the RW approach on HP. For future prospective studies, a distinction between the RW approach and the extended RW approach is necessary to identify a true difference between the RW approach and the other approaches by which the cochlea is accessed through various degrees of drilling and subsequent breach of the endosteal endoscalar lining.

### Considerations Following the SR

Medium flexible electrodes are well studied and approved for EAS surgery in the case of partial deafness. However, even though a high HP rate at 12 months is reported, patients may progressively lose their residual hearing over time and sometimes immediately after surgery, necessitating a switch from EAS to full electric stimulation. This leaves the patient without electric stimulation in the second cochlear turn, which means that there is no stimulation of around 25% of the spiral ganglion located within the second turn, which is an inefficient use of the CI.

Conversely, this SR revealed a high HP rate with the long flexible electrodes, which was not statistically different from the HP outcomes with the medium flexible electrodes. Long flexible electrodes offer the advantage of providing electrical stimulation to the full spiral ganglion in case of future deterioration of the residual hearing.

The data of this SR support the concept presented by the Japanese CI team of Usami SI (7), who proposed choosing a longer electrode, in particular, the 28-mm-long electrodes, when implanting a patient with residual hearing.

Therefore, longer flexible electrodes should be considered equally for EAS surgery. The authors advocate the use of these longer electrodes to reach the second cochlear turn, in particular when a stable hearing was not ascertained or uncertain in the next years.

### Future Studies

This SR reveals the necessity of prospective studies assessing HP surgery with deep 650° electrode insertions in patients with RH. These studies should distinguish between atraumatic and traumatic insertion approaches (RW vs. extended RW/cochleostomy) and factors in cochlear duct length measurements. Current studies report that new otosurgical planning software (Otoplan) is suitable for cochlear size calculations (59) and cochlear segmentation algorithms will further improve the cochleometric data. As reported in earlier studies (Figure 1), the use of a 28-mm-long electrode can be advocated in the majority of cases. Preliminary data do not demonstrate any apparent adverse effects of a full overlap between the acoustic and electric frequencies (60, 61).

## Conclusion

Both medium-length and longer electrode arrays showed high hearing preservation rates. Considering the hearing deterioration over time, implanting a longer electrode at primary surgery should be considered, thus preventing the need for future reimplantation.

## Data Availability

The original contributions presented in the study are included in the article/Supplementary Material; further inquiries can be directed to the corresponding author/s.

## Appendix

**Appendix 1:** Comprehensive hearing preservation and electric-acoustic stimulation bibliography.

Bibliography
FLEX24 group
1. Gsötttner W, Kiefer J, Baumgartner W, Pok S, Peters S, Adunka O. Hearing preservation in cochlear implantation for electric acoustic stimulation. *Acta Otolaryngol*. (2004) **124**:348–352.
2. Adunka OF, Pillsbury HC, Adunka MC, Buchman CA. Is electric acoustic stimulation better than conventional cochlear implantation for speech perception in quiet? *Otol Neurotol*. (2010 Sep) **31**(7):1049–54.
3. Arnoldner C, Gstoettner W, Riss D, Wagenblast J, Honeder C, Blineder M, et al. Residual hearing preservation using the suprameatal approach for cochlear implantation. *Wien Klin Wochenschr*. (2011 Oct) **123**(19-20):599–602.
4. Helbig S, Van de Heyning P, Kiefer J, Baumann U, Kleine-Punte A, Brockmeier H, et al. Combined electric acoustic stimulation with the PULSARCI (100) implant system using the FLEX(EAS) electrode array. *Acta Otolaryngol*. (2011 Jun) **131**(6):585–95.
5. Erixon E, Köbler S, Rask-Andersen H. Cochlear implantation and hearing preservation: Results in 21 consecutively operated patients using the round window approach. *Acta Otolaryngol*. (2012 Sep) **132**(9):923–31.
6. Tamir S, Ferrary E, Borel S, Sterkers O, Bozorg Grayeli A. Hearing preservation after cochlear implantation using deeply inserted flex atraumatic electrode arrays. *Audiol Neurootol*. (2012) **17**(5):331–7.
7. Rajan GP, Kuthubutheen J, Hedne N, Krishnaswamy J. The role of preoperative, intratympanic glucocorticoids for hearing preservation in cochlear implantation: a prospective clinical study. *Laryngoscope*. (2012 Jan) **122**(1):190–5. doi: 10.1002/lary.22142.
8. de Carvalho GM, Guimaraes AC, Duarte AS, Muranaka EB, Soki MN, Martins RS, et al. Hearing Preservation after cochlear implantation: UNICAMP Outcomes. *Int J Otolaryngol*. (2013) **2013**:107186.
9. Nordfalk KF, Rasmussen K, Hopp E, Greisiger R, Jablonski GE. Scalar position in cochlear implant surgery and outcome in residual hearing and the vestibular system. *Int J Audiol*. (2014 Feb) **53**(2):121–7.
10. Santa Maria PL, Domville-Lewis C, Sucher CM, Chester-Browne R, Atlas MD. Hearing preservation surgery for cochlear implantation–hearing and quality of life after 2 years. *Otol Neurotol*. (2013 Apr) **34**(3):526–31.
11. Adunka OF, Dillon MT, Adunka MC, King ER, Pillsbury HC, Buchman CA. Hearing preservation and speech perception outcomes with electric-acoustic stimulation after 12 months of listening experience. *Laryngoscope*. (2013 Oct) **123**(10):2509–15.
12. Mertens G, Punte AK, Cochet E, De Bodt M, Van de Heyning P. Long-term follow-up of hearing preservation in electric-acoustic stimulation patients. *Otol Neurotol*. (2014 Dec) **35**(10):1765–72.
13. Usami S, Moteki H, Tsukada K, Miyagawa M, Nishio SY, Takumi Y, et al. Hearing preservation and clinical outcome of 32 consecutive electric acoustic stimulation (EAS) surgeries. *Acta Otolaryngol*. (2014 Jul) **134**(7):717–27.
14. Guimarães AC, Carvalho GM, Duarte AS, Bianchini WA, Sarasty AB, Gregorio MF, et al. Hearing preservation and cochlear implants according to inner ear approach: multicentric evaluation. *Braz J Otorhinolaryngol*. (2015 Mar-Apr) **81**(2):190–6.
15. Bruce IA, Felton M, Lockley M, Melling C, Lloyd SK, Freeman SR, et al. Hearing preservation cochlear implantation in adolescents. *Otol Neurotol*. (2014 Oct) **35**(9):1552–9.
16. Moteki H, Kitoh R, Tsukada K, Iwasaki S, Nishio SY, Usami S. The advantages of sound localization and speech perception of bilateral electric acoustic stimulation. *Acta Otolaryngol*. (2015 Feb) **135**(2):147–53.
17. Mahmoud AF, Massa ST, Douberly SL, Montes ML, Ruckenstein MJ. Safety, efficacy, and hearing preservation using an integrated electro-acoustic stimulation hearing system. *Otol Neurotol*. (2014 Sep) **35**(8):1421–5.
18. Suhling MC, Majdani O, Salcher R, Leifholz M, Büchner A, Lesinski-Schiedat A, et al. The Impact of Electrode Array Length on Hearing Preservation in Cochlear Implantation. *Otol Neurotol*. (2016 Sep) **37**(8):1006–15.
19. Pillsbury 3rd HC, et al. Multicenter US Clinical Trial with an Electric-Acoustic Stimulation (EAS) System in Adults: Final Outcomes. *Otol Neurotol.* (2018 Mar) **39**(3):299–305.
20. Rader T, Bohnert A, Matthias C, Koutsimpelas D, Kainz MA, Strieth S. Hearing preservation in children with electric-acoustic stimulation after cochlear implantation: Outcome after electrode insertion with minimal insertion trauma. *HNO*. (2018 Jul) **66**(Suppl 2):56–62.
21. Skarzynski PH, Skarzynski H, Dziendziel B, Rajchel JJ, Gos E, Lorens A. Hearing Preservation with the Use of Flex20 and Flex24 Electrodes in Patients with Partial Deafness. *Otol Neurotol*. (2019 Oct) **40**(9):1153–1159. doi: 10.1097/MAO.0000000000002357. 72.
22. Thompson NJ, Dillon MT, Bucker AL, King ER, Pillsbury 3rd HC, Brown KD. Electric-Acoustic Stimulation After Reimplantation: Hearing Preservation and Speech Perception. *Otol Neurotol*. (2019 Feb) **40**(2):e94–e98.
23. Skarzynski H, Lorens A, Dziendziel B, Rajchel JJ, Matusiak M, Skarzynski PH. Electro-Natural Stimulation in Partial Deafness Treatment of Adult Cochlear Implant Users: Long-Term Hearing Preservation Results. *ORL J Otorhinolaryngol Relat Spec*. (2019) **81**(2-3):63–72.
24. Schart-Morén N, Erixon E, Li H, Rask-Andersen H. Cochlear implantation, and residual hearing preservation long-term follow-up of the first consecutively operated patients using the round window approach in Uppsala, Sweden. *Cochlear Implants Int*. (2020 Sep) **21**(5):246–259.
25. Sprinzl GM, Schoerg P, Edlinger SH, Magele A. Long-term Hearing Preservation in Electric Acoustic Cochlear Implant Candidates. *Otol Neurotol*. (2020 Jul) **41**(6):750–757. doi: 10.1097/MAO.0000000000002627.
FLEX28/FLEXSOFT group
26. Helbig S, Baumann U, Hey C, Helbig M. Hearing preservation after complete cochlear coverage in cochlear implantation with the free-fitting FLEXSOFT electrode carrier. *Otol Neurotol*. (2011 Aug) **32**(6):973–9.
27. Bruce IA, Bates JE, Melling C, Mawman D, Green KM. Hearing preservation via a cochleostomy approach and deep insertion of a standard-length cochlear implant electrode. *Otol Neurotol*. (2011 Dec) **32**(9):1444–7.
28. Skarzynski H, Lorens A, Zgoda M, Piotrowska A, Skarzynski PH, Szkielkowska A. Atraumatic round window deep insertion of cochlear electrodes. *Acta Otolaryngol*. (2011 Jul) **131**(7):740–9.
29. Rogers B, Prentiss S, Staecker H. Expanding cochlear implantation to patients with residual mid and high frequency hearing. *Otorinolaringologia*. (2012 Dec) **62**(4):183–90.
30. Jayawardena J, Kuthubutheen J, Rajan G. Hearing preservation and hearing improvement after reimplantation of pediatric and adult patients with partial deafness: a retrospective case series review. *Otol Neurotol*. (2012 Jul) **33**(5):740–4.
31. Nordfalk KF, Rasmussen K, Hopp E, Bunne M, Silvola JT, Jablonski GE. Insertion Depth in Cochlear Implantation and Outcome in Residual Hearing and Vestibular Function. *Ear Hear*. (2016 Mar-Apr) **37**(2):e129–37.
32. Jones HAS, Powell HRF, Hall A, Lavy J, Shaida A, Saeed S, et al. Evaluating inter-aural hearing preservation in bilateral paediatric cochlear implantation. *Cochlear Implants Int*. (2018 Nov) **19**(6):307–311.
33. Moteki H, Nishio SY, Miyagawa M, Tsukada K, Noguchi Y, Usami SI. Feasibility of hearing preservation for residual hearing with longer cochlear implant electrodes. *Acta Otolaryngol*. (2018 Dec) **138**(12):1080–1085.
34. Sierra C, Calderón M, Bárcena E, Tisaire A, Raboso E. Preservation of Residual Hearing After Cochlear Implant Surgery with Deep Insertion Electrode Arrays. *Otol Neurotol*. (2019 Apr) **40**(4):e373–e380.
35. Yoshimura H, Moteki H, Nishio SY, Usami SI. Electric-acoustic stimulation with longer electrodes for potential deterioration in low-frequency hearing. *Acta Otolaryngol.* (2020 Aug) **140**(8):632–638.
